# CFANet: The Cross-Modal Fusion Attention Network for Indoor RGB-D Semantic Segmentation

**DOI:** 10.3390/jimaging11060177

**Published:** 2025-05-27

**Authors:** Long-Fei Wu, Dan Wei, Chang-An Xu

**Affiliations:** 1 School of Mechanical and Automotive Engineering, Shanghai University of Engineering Science, Shanghai 201620, China; longfei077167@163.com; 2 College of Materials and Energy, South China Agricultural University, Guangzhou 510642, China; xuca2020@163.com

**Keywords:** cross-modal fusion, RGB-D, feature extraction, feature interaction

## Abstract

Indoor image semantic segmentation technology is applied to fields such as smart homes and indoor security. The challenges faced by semantic segmentation techniques using RGB images and depth maps as data sources include the semantic gap between RGB images and depth maps and the loss of detailed information. To address these issues, a multi-head self-attention mechanism is adopted to adaptively align features of the two modalities and perform feature fusion in both spatial and channel dimensions. Appropriate feature extraction methods are designed according to the different characteristics of RGB images and depth maps. For RGB images, asymmetric convolution is introduced to capture features in the horizontal and vertical directions, enhance short-range information dependence, mitigate the gridding effect of dilated convolution, and introduce criss-cross attention to obtain contextual information from global dependency relationships. On the depth map, a strategy of extracting significant unimodal features from the channel and spatial dimensions is used. A lightweight skip connection module is designed to fuse low-level and high-level features. In addition, since the first layer contains the richest detailed information and the last layer contains rich semantic information, a feature refinement head is designed to fuse the two. The method achieves an mIoU of 53.86% and 51.85% on the NYUDv2 and SUN-RGBD datasets, which is superior to mainstream methods.

## 1. Introduction

Semantic segmentation for indoor scenarios is a dense prediction task that aims to assign a category label to each pixel in an image. It is applied in medical image analysis, industrial robots, intelligent driving, and other fields [1]. To enhance the accuracy of semantic segmentation, convolutional neural network (CNN) [2] is commonly employed by researchers for the extraction of image features. The powerful feature extraction ability of CNNs has led to notable advances in segmentation. For example, He et al. [3] propose a semantic segmentation model based on pyramid scene parsing, which is characterized by combining kernels of different sizes to create a spatial pyramid pooling network. PointFlow [4] is proposed by Huang et al. This method adaptively utilizes high-semantic low-resolution feature maps to enhance low-semantic high-resolution feature maps, thereby obtaining feature maps with both high semantic information and high resolution. Although CNN shows a strong performance in information representation, RGB images are a planarization of 3D space and lose depth information. With the progression in depth camera technology, depth maps are progressively being employed by researchers as a supplementary data resource for the execution of image semantic segmentation. This leads to the emergence of the semantic segmentation of RGB and depth images (RGB-D). Addressing the semantic gap between RGB and depth images and reducing the loss of detail information are current hot topics in RGB-D semantic segmentation [5].

**Figure 1:**
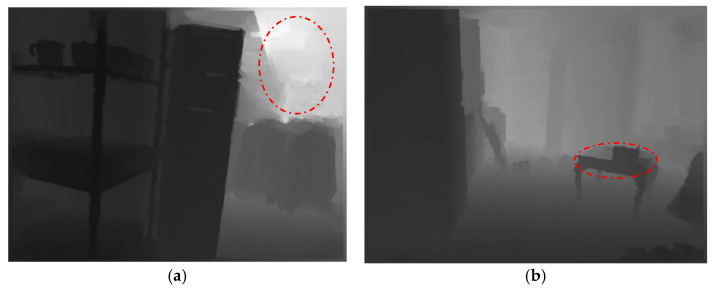
Noise in depth maps. (**a**) Type one noise: As shown in the red box, the boundary in the distance is not clear. (**b**) Type two noise: As shown in the red box, different objects at the same distance from the camera may be segmented as a single object.

RGB images may generate noise due to the similarity of texture features between different objects. In contrast, depth maps can provide relative distance information of objects, which is not affected by the interference of similar colors and textures of objects and can effectively distinguish the relative positions of occluded objects. Therefore, the information provided by depth maps can compensate for the deficiencies of indoor RGB images in terms of occlusion and similar textures. However, depth maps themselves may also have noise. For instance, as depicted in Figure 1a, limitations in camera hardware can result in the blurring of object boundaries at a distance, which in turn may lead to the incorporation of noise during the process of boundary information extraction. Also, as shown in Figure 1b, different objects at the same distance from the camera may be incorrectly segmented as a single object. To bridge the semantic gap between RGB images and depth maps, researchers are exploring various multi-modal fusion methods.

RGB images may generate noise due to similar textures among different objects. However, depth maps can provide relative distance information of objects and are not affected by the color and texture of similar objects. The information provided by depth maps can compensate for the shortcomings of indoor RGB images, such as occlusions and similar textures.

**Figure 2:**
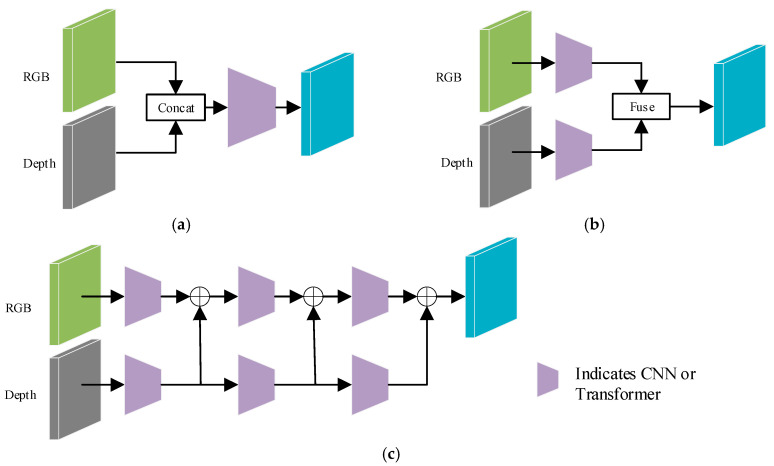
Fusion methods. (**a**) Early fusion. (**b**) Late fusion. (**c**) Multi-level fusion.

As shown in Figure 2, depending on the timing of fusion between RGB and depth features, fusion methods can be categorized into three types: (1) Early fusion: This approach first concatenates the RGB and depth images and then generates feature maps through convolutional layers. This fusion strategy is pioneered in the early work of Couprie et al. [6], effectively avoiding the reliance on handcrafted features in RGB-D semantic segmentation tasks. However, the semantic segmentation performance of this early fusion approach is frequently limited due to the existence of a semantic gap between RGB and depth images. (2) Late fusion: This fusion strategy employs a dual-branch network architecture, where the two branches independently extract features from the RGB images and depth maps, respectively, and fuse the extracted feature at the later stages of the network. For example, Yang et al. [7] apply Uniformer [8] as the backbone network to separately extract features from RGB and depth images and utilize a graph reasoning module to effectively fuse the output features of the two branches. (3) Multi-level fusion: This strategy adopts a progressive layer-by-layer approach to fuse RGB and depth features and enhances the complementarity between the two modalities through a dynamic weight configuration mechanism. For instance, Kazerouni et al. [9] propose an RGB-D image semantic segmentation network based on multi-modal image feature fusion, which gradually merges RGB and depth image features at different levels using a direct summation method. Although methods of types (b) and (c) have demonstrated certain improvements in RGB-D semantic segmentation, the exploration of the intrinsic correlations and interdependencies between RGB and depth data still presents a considerable area for future research.

The semantic gap between RGB and depth images can also be effectively narrowed through the design of appropriate feature extraction modules. This is attributed to the capability of well-designed feature extraction modules to suppress noise in the extracted feature representations [7]. Many studies [10,11] adopt similar methods to extract features from both RGB and depth images, or merely use depth images as a supplement to RGB images, overlooking the differences and complementarities between these two modalities. For instance, Zhou et al. [12] focus on designing modules to enhance the fusion process and employ a feature refinement module to extract semantic features. However, this approach does not sufficiently emphasize the initial feature extraction stage. Considering the modality differences between RGB and depth images, two distinct feature extraction modules are designed: the RGB Feature Extraction Module (BFEM) and the Depth Feature Extraction Module (DFEM). BFEM incorporates asymmetric convolutions based on ASPP [13] to capture features in horizontal and vertical spatial directions, and integrates a cross-shaped attention mechanism to obtain contextual information from global dependencies. In contrast, DFEM extracts prominent unimodal features from depth images in both channel and spatial dimensions. Considering the evident semantic gap between these two modalities, an Adaptive Feature Complementary Fusion Module (AFFCM) is proposed for automatically aligning the features of these two modalities.

As convolutional neural networks deepen, there is a possibility of losing useful information, which is another research hotspot in the field of semantic segmentation. Multi-level contextual information is crucial for multi-scale object segmentation: low-level contextual information contains rich details of object boundaries, while high-level contextual information encodes relationships between different objects. To fully capture the features of multi-scale objects in images, researchers conduct extensive studies and propose many classic network models, such as image pyramid [14], feature pyramid [15], and spatial pyramid pooling models [3]. These methods aim to utilize the key detailed information provided by low-level features, such as texture and spatial relationships, to better capture the features of multi-scale objects. Additionally, the feature maps output by the first layer of the encoder retain detailed internal information but lack semantic content, while the feature maps output by the last layer of the decoder contain rich semantic information but lack detailed information [16]. A common practice in previous studies is to perform additive operations on these two types of feature maps. Although this method reduces computational complexity, it often leads to a decrease in semantic segmentation accuracy. To address this issue, we design the feature refinement head (FRH) to refine feature maps from both spatial and channel dimensions. Furthermore, the skip connection module (SCM) is proposed for fusing the feature information of multi-scale features.

To address the challenges of semantic gaps and the loss of detailed information in RGB-D, this paper presents the following contributions:

(1) We introduce a novel dual-branch RGB-D semantic segmentation network named the Cross-Modal Fusion Attention Network (CFANet). Tailored feature extraction modules are designed based on the distinct characteristics of RGB and depth maps, subsequently enhancing segmentation accuracy through adaptive cross-modal feature fusion.
(2) BFEM introduces asymmetric convolution on the basis of dilated convolution to alleviate the gridding effect of dilated convolution. Additionally, it achieves rich contextual learning through dense connections and criss-cross attention. DFEM extracts significant unimodal features from both the channel and spatial dimensions of depth maps.
(3) We guide the RGB branch and depth map branch to interact rather than simply treating the depth map as a complement to the RGB image.
(4) AFFCM employs a multi-head self-attention mechanism to address the semantic discrepancies between RGB and depth map features, resulting in their adaptive alignment. This process effectively enhances the complementary information exchange between the two modalities and mitigates redundancy.
(5) We adopt different strategies for feature maps of different scales. Multi-scale feature maps are fused through SCM; considering that the first layer contains the richest detailed information and the last layer contains the richest semantic information, we designed FRH to fuse both.

## 2. Related Works

Attention-based RGB-D semantic segmentation methods demonstrate significant advantages in indoor scene understanding [17]. RGB images and depth maps capture texture–color features and geometric–distance features, respectively, and how to fuse them efficiently through the attention mechanism remains a key challenge in improving segmentation accuracy. The existing research primarily focuses on two directions: optimizing single-modal attention mechanisms and developing cross-modal feature alignment strategies. The technical evolution of these approaches is systematically reviewed from a methodological perspective.

### 2.1. Optimization of Single-Modal Attention Mechanisms

In RGB-D segmentation, single-modal attention mechanisms are primarily used to enhance the representation of key features within each modality. In spatial attention, Huang et al. proposed criss-cross attention [18], which models long-range dependencies along horizontal and vertical directions, effectively preserving structural information in depth maps; Woo et al.’s CBAM module [19] employs a spatial attention module (SAM) to focus on key regions, but its fixed weight allocation strategy is less adaptable to dynamic scene changes. In channel attention research, SENet [20] captures inter-channel dependencies via global average pooling, while the Efficient Channel Attention model [21] replaces dimensionality reduction with a 1D convolution, enhancing the computational efficiency of channel attention. Notably, these methodologies are devised for the enhancement of unimodal features, with the comprehensive exploration of synergistic relationships among cross-modal features remaining insufficient.

### 2.2. Cross-Modal Feature Alignment Strategies

To address the heterogeneity of RGB-D data, various cross-modal alignment methods have been proposed. The initial methodologies predominantly employ feature concatenation or weighted fusion strategies; however, such approaches frequently lead to information redundancy and face challenges in effectively bridging the semantic gap across different modalities. With the development of attention mechanisms, attention-based alignment methods have become mainstream: A feature decoupling and interaction mechanism is proposed by Hu et al. [22], where cross-modal feature transfer is enabled through gating modules; A spatial pyramid structure is employed by Fu et al. [23] for multi-scale feature fusion, where feature representation is enhanced through hierarchical receptive fields, but real-time applications are limited by their high computational complexity. Recently, multi-head attention mechanisms [24] have been introduced to cross-modal alignment, achieving flexible and accurate alignment through learnable projection matrices for feature space mapping. Current methods demonstrate significant progress in cross-modal alignment by enhancing fusion capabilities across different scales through spatial pyramid structures [25] or sliding window mechanisms [26], and by dynamically weighting features between modalities via gating mechanisms [27] or self-attention [24,28], thereby more effectively capturing the complementary relationships between RGB images and depth maps.

Building upon these outstanding research contributions, we construct CFANet. In CFANet, we integrate criss-cross attention [18] into BFEM. DFEM utilizes attention mechanisms to extract significant unimodal features from the spatial and channel dimensions of the depth map. AFCFM employs multi-head attention to align RGB features and depth map features. Benefiting from attention mechanisms, the complementary information between dual modalities is effectively enhanced while redundant information is suppressed by AFCFM.

## 3. Method

### 3.1. Overview of the Architecture

**Figure 3:**
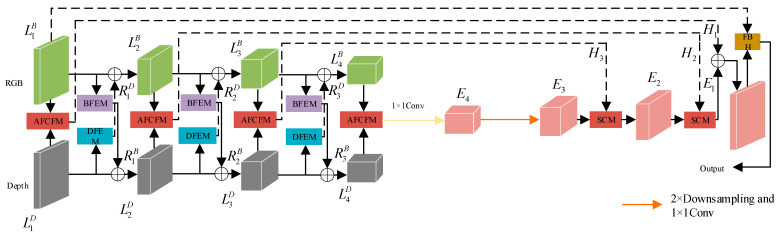
Overall architecture of CFANet.

Figure 3 presents the framework of the proposed CFANet, which is constructed upon a conventional encoder–decoder architecture for the purpose of achieving end-to-end semantic segmentation. During the encoding phase, RGB images and depth maps are input separately into their respective network branches. In both branches, we employ ResNet50 [20] to extract features from the images, yielding four feature maps at different resolutions for each type of image. $L_{i}^{B}$ (i = 1, 2, 3, 4) represents the extracted RGB image features, and $L_{i}^{D}$ represents the extracted depth image features. BFEM is designed to develop RGB image features enriched with contextual information, while DFEM focuses on capturing significant unimodal features from the depth map. Applying BFEM to $L_{i}^{B}$ results in the re-extracted feature maps $R_{i}^{B}$. The addition of $R_{i}^{B}$ and $L_{i}^{D}$ produces new feature maps $L_{i+1}^{D}$, and the addition of $R_{i}^{D}$ and $L_{i}^{B}$ produces a new feature map $L_{i+1}^{B}$, facilitating feature interaction. This process is illustrated by the following Formula (1):

(1) $$\left\{\begin{array}{l} L_{i+1}^{D}=L_{i}^{D}+R_{i}^{B}\\ L_{i+1}^{B}=L_{i}^{B}+R_{i}^{D} \end{array}\right.,(i=1,2,3)$$

As illustrated in Figure 3, to mitigate the semantic gap present in different modal images, AFCFM is employed to merge feature maps from different modalities, resulting in the fused features $H_{i}$, as described in the following text. The SCM designed in this paper strikes a good balance between accuracy and computational efficiency. Considering the unique characteristics of $E_{1}$ and $L_{1}^{B}$, FRH is developed. FRH further refines the feature maps from both spatial and channel perspectives.

### 3.2. RGB Feature Extraction Module and Depth Map Feature Extraction Module

The modal differences between RGB images and depth maps are primarily manifested in the way information is expressed. Rich information about object color, texture, and appearance is provided by RGB images, while the distance or depth information of objects is provided by depth maps. Owing to the distinct information provided by these two modalities, effectively combining them poses a significant challenge in RGB-D semantic segmentation tasks. To tackle this issue, two feature extraction modules are meticulously crafted to align with the unique attributes of each type of data.

#### 3.2.1. RGB Feature Extraction Module

Indoor RGB images exhibit the following characteristics: (1) Indoor scenes comprise objects of various scales, such as small-sized decorations and large-sized wardrobes. (2) Indoor image backgrounds are complex, potentially containing numerous background objects similar to the target objects. To address these challenges, there is a need to increase the receptive field and learn rich contextual information. In previous research, dilated convolutions were introduced to enlarge the receptive field without introducing a large number of parameters. However, dilated convolutions may lead to a loss of correlation between adjacent pixels. BFEM not only alleviates the aforementioned challenges but also enables the learning of abundant contextual information.

**Figure 4:**
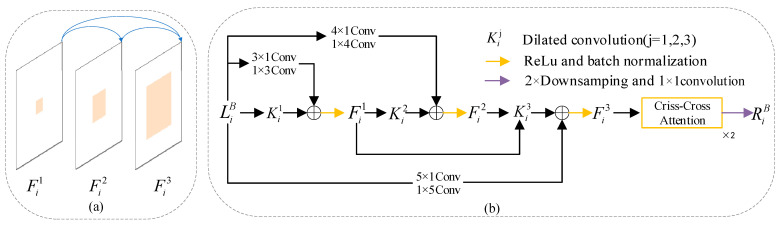
Detailed structure of the proposed BFEM. (**a**) Diagram of dense connection. (**b**) Flowchart of BFEM.

To mitigate the gridding issue associated with dilated convolutions, BFEM incorporates asymmetric convolutions [29]. The structure of BFEM is illustrated in Figure 4. After filtering the feature map $L_{i}^{B}$ with dilated convolutions, we immediately fuse it with the feature map processed by the corresponding asymmetric convolutions. Additionally, we sum up the previously processed feature maps to enhance dense connections, as shown in Figure 4a. Dense connections allow for the repeated reuse of signals, providing substantial expansion of the receptive field and enabling the learning of more contextual information [22]. We implement the above processes using the following Formula (2):

(2) $$\left\{\begin{array}{l} F_{i}^{1}=\delta \left[\left(L_{i}^{B}⊗K_{i}^{1}\right)+C_{1}\left(L_{i}^{B}\right)\right]\\ F_{i}^{2}=\delta \left[\left(F_{i}^{1}⊗K_{i}^{2}\right)+C_{2}\left(L_{i}^{B}\right)\right]\\ F_{i}^{3}=\delta \left[\left(F_{i}^{1}⊗K_{i}^{3}\right)+\left(F_{i}^{2}⊗K_{i}^{3}\right)+C_{3}\left(L_{i}^{B}\right)\right] \end{array}\right.$$

In Formula (2), $F_{i}^{m}$ (m=1,2,3; i=1,2,3) represents the feature map generated after each densely connected operation; $K_{i}^{1}$ represents the dilated convolution with a dilation rate of 1, $K_{i}^{2}$ represents the dilated convolution with a dilation rate of 2, and $K_{i}^{3}$ represents the dilated convolution with a dilation rate of 3; $C_{1}$ represents a 3 × 1 convolution followed by a 1 × 3 convolution, $C_{2}$ represents a 4 × 1 convolution followed by a 1 × 4 convolution, and $C_{3}$ represents a 5 × 1 convolution followed by a 1 × 5 convolution; $F_{i}^{B}$ (i=1,2,3) represents the feature map generated by the *i*-th layer in the RGB network branch; δ is a 2× downsampling followed by a 1 × 1 convolution.

To effectively capture global contextual information while minimizing computational overhead, criss-cross attention is employed [19]. The feature map $F_{i}^{3}$ undergoes two criss-cross attention operations to yield $R_{i}^{B}$, as shown in Figure 4b.

#### 3.2.2. Depth Map Feature Extraction Module

Indoor depth maps may contain noise, especially in regions with uneven lighting or texture absence. Consequently, features provided by the depth map may introduce information detrimental to segmentation accuracy. To alleviate this issue, we employ DFEM to extract significant unimodal features in both spatial and channel dimensions.

**Figure 5:**
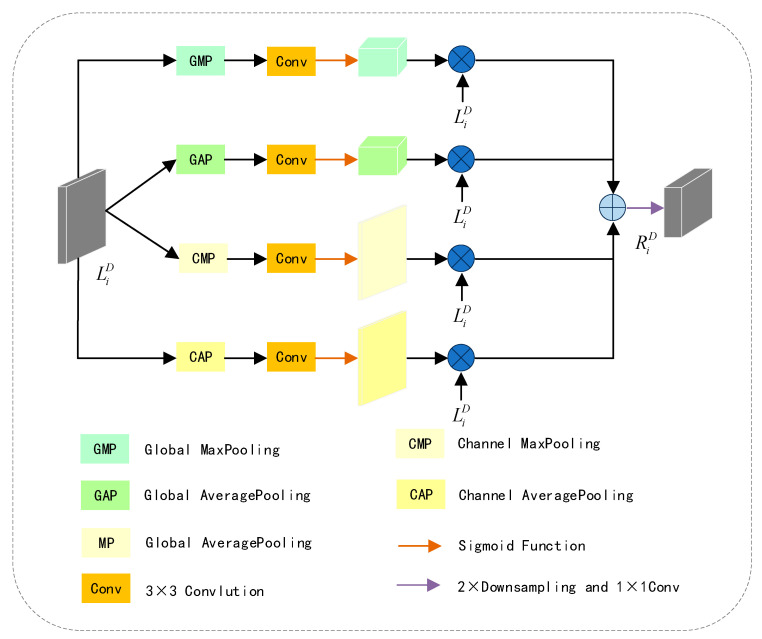
Detailed structure of the proposed DFEM.

The structure of DFEM is illustrated in Figure 5. The two upper branches extract weighted features of $L_{i}^{D}$ from the spatial dimension, while the two lower branches extract weighted features of $L_{i}^{D}$ from the channel dimension. Specifically, the two upper branches take the feature maps processed by global max pooling and global average pooling, input them into a 3 × 3 convolution for adaptive parameter updates, apply a sigmoid function to obtain spatial feature weights, and finally perform element-wise multiplication of the spatial feature weights with the input feature $L_{i}^{D}$ to obtain spatial-weighted features. Similarly, the two lower branches take the feature maps processed by channel max pooling and channel average pooling, input them into a 3 × 3 convolution for adaptive parameter updates, apply a sigmoid function to obtain channel feature weights, and finally perform element-wise multiplication of the channel feature weights with the input feature $L_{i}^{D}$ to obtain channel-weighted features. The spatial-weighted features and channel-weighted features are added, and after 2× downsampling followed by a 1 × 1 convolution to adjust the channel number, the result $R_{i}^{D}$ is obtained. This process is illustrated by the following Formula (3):

(3) $$R_{i}^{D}=\delta \left\{\sum_{P=1}^{4}\left[\alpha \left(C\mathrm{onv}(P(L_{i}^{D}))\right)⊗L_{i}^{D}\right]\right\}$$

In Formula (3), when P=1, P represents global max pooling; when P=2, P represents global average pooling; when P=3, P represents channel max pooling; and when P=4, P represents channel average pooling. ⊗ denotes element-wise multiplication, Conv signifies a 3 × 3 convolution, α is the sigmoid function, and δ represents 2× downsampling followed by a 1 × 1 convolution.

### 3.3. Adaptive Feature Complementary Fusion

The feature maps extracted from RGB and depth images not only contain complementary information but also entail redundant details. Striking a balance between enhancing complementary information and reducing redundancy is our objective. Leveraging the potent feature representation capabilities of multi-head attention [30], which empowers the model to learn relationships between different parts and representations effectively, AFCFM utilizes a multi-head attention mechanism to adaptively align the feature maps generated from RGB and depth images. Subsequently, it enhances the representation of complementary information from both channel and spatial perspectives, mitigating redundancy.

**Figure 6:**
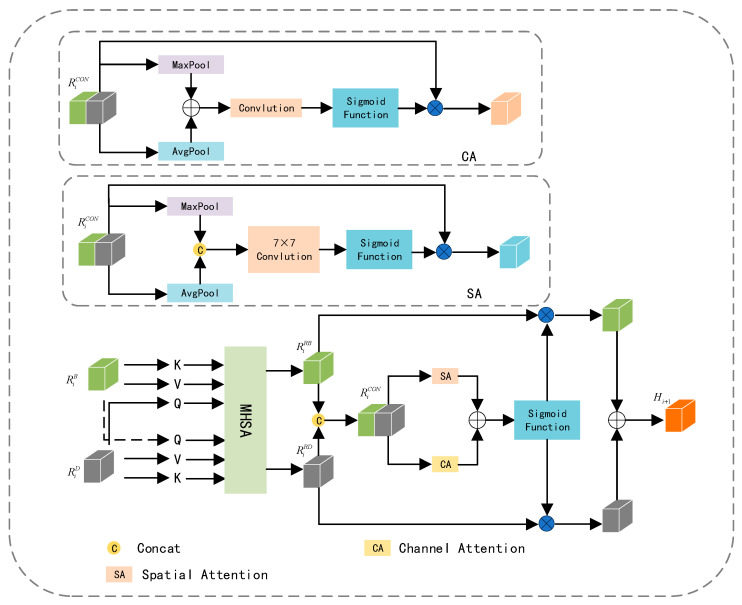
Detailed structure of the proposed AFCFM.

As illustrated in Figure 6, the feature map $R_{i}^{B}$ obtained from the RGB image branch and the feature map $R_{i}^{D}$ obtained from the depth image branch are linearly mapped to derive $Q^{B},K^{B},V^{B}$ and $Q^{D},K^{D},V^{D}$. It is noteworthy that, to align $R_{i}^{B}$ and $R_{i}^{D}$, this paper uses $Q^{D}$ as the input to the multi-head self-attention mechanism processing $R_{i}^{B}$, and employs $Q^{B}$ as the input to the distinct multi-head self-attention mechanism handling $R_{i}^{D}$. This process can be described by the following Formula (4):

(4) $$\left\{\begin{array}{l} R_{i}^{RB}=MHSA(Q^{D},K^{B},V^{B})\\ R_{i}^{RD}=MHSA(Q^{B},K^{D},V^{D}) \end{array}\right.$$

In Formula (4), MHSA represents multi-head attention. $Q^{B},K^{B},V^{B}$ is the vector obtained from the RGB image, and $Q^{D},K^{D},V^{D}$ is the vector obtained from the depth image. $R_{i}^{RB}$ represents the aligned RGB feature map, and $R_{i}^{RD}$ represents the aligned depth feature map after the alignment process.

Influenced by the idea of the attention mechanism, we assign different weights to $R_{i}^{RB}$ and $R_{i}^{RD}$ to enhance complementary information while reducing redundancy. In specific experiments, we adopt the framework of CBAM [19] and apply the attention mechanism separately in the channel and spatial dimensions. However, the original channel attention mechanism in CBAM employs two consecutive fully connected layers, and the dimension reduction operation in it has a negative impact on the prediction of channel attention, resulting in a lower computational efficiency. We replace this with a simple one-dimensional convolution to adaptively explore the weights for each channel. The channel attention module’s structure is represented as CA in Figure 6, and the structure of spatial attention is denoted as SA. The weight information obtained from CA and SA is then mapped to $R_{i}^{RB}$ and $R_{i}^{RD}$, and the fused feature $H_{i+1}$ is derived through addition. This process can be described by the following Formulas (5) and (6):

(5) $$R_{i}^{CON}=C\mathrm{on}cat(R_{i}^{RB},R_{i}^{RD})$$

(6) $$H_{i+1}=\alpha \left[SA(R_{i}^{CON})+CA(R_{i}^{CON})\right]⊗R_{i}^{RB}+\alpha \left[SA(R_{i}^{CON})+CA(R_{i}^{CON})\right]⊗R_{i}^{RD}$$

In the formulas, $E_{i-1}=PS\{\alpha \left[Conv(Con(E_{i},H_{i}))\right]⊗Con(E_{i},H_{i})\}$ represents spatial attention, CA represents channel attention. α is the sigmoid function, ⊗ is element-wise multiplication. $H_{i+1}$ is the fused feature map obtained after the attention mechanisms.

### 3.4. Skip Connection Module and Feature Refinement Head

The feature maps generated by the encoder retain rich detailed information but lack semantic content. Conversely, those generated by the decoder contain abundant semantic information but suffer from significant information loss as the network deepens. In prior work, it has been common practice to perform a simple addition operation on these two feature maps. While this strategy is computationally favorable, it leads to a degradation in semantic segmentation performance. To address this issue, we propose two effective modules, namely SCM and RFH, which enhance feature fusion while maintaining computational efficiency.

#### 3.4.1. Feature Refinement Head

**Figure 7:**
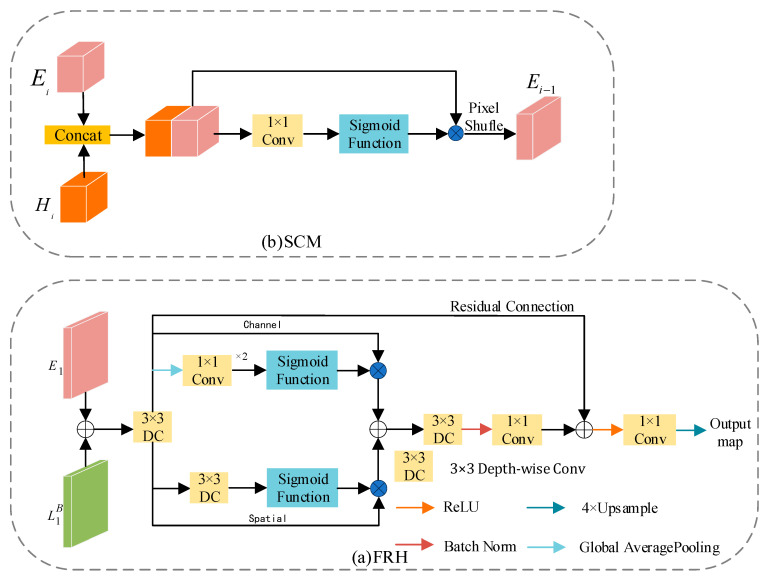
Detailed structure of the proposed FRH and SCM. (**a**) Architectural diagram of FRH. (**b**) Architectural diagram of SCM.

The structure of FRH is illustrated in Figure 7a. The FRH module aims to deeply fuse $L_{1}^{B}$ and $E_{1}$, maximizing the utilization of both detailed and semantic information. Building upon feature fusion, the feature maps undergo refinement through spatial and channel branches. Specifically, the spatial branch employs 3 × 3 depthwise separable convolutions to generate attention maps, aiming to reduce parameter count and enhance computational efficiency. The channel branch, on the other hand, first compresses the feature maps into a one-dimensional vector using global average pooling. It then utilizes 1 × 1 convolutions to reduce the number of channels to one-fourth of the original, followed by another 1 × 1 convolution to restore it to the original channel count. This operation is intended to increase network depth while mitigating the risk of overfitting. Ultimately, the feature maps generated by the spatial and channel paths are further fused through summation. Additionally, to prevent network degradation during training, this paper introduces a residual connection mechanism, enhancing the stability and effectiveness of network training.

#### 3.4.2. Skip Connection Module

In previous studies, a common practice involves the use of simple addition operations on feature maps or the employment of complex modules to fuse feature maps from the decoding and encoding stages. Unlike previous research, concatenation is utilized to combine the feature maps input to the SCM, thereby obtaining features with a higher representational capacity. In contrast to more complex models, we use only a 1 × 1 convolution to update parameters. Additionally, SCM employs Pixel Shuffle [31] to reorganize channels, enabling an increase in feature map resolution and the retention of more detailed information without introducing new parameters. The structure of SCM is illustrated in Figure 7b. This process can be described by the following Formula (7):

(7) $$E_{i-1}=PS\{\alpha \left[Conv(Con(E_{i},H_{i}))\right]⊗Con(E_{i},H_{i})\}$$

where PS denotes Pixel Shuffle, Con represents Concat operation, α is the sigmoid function, and ⊗ is element-wise multiplication. Conv signifies a 1 × 1 convolution.

## 4. Experiment

### 4.1. Dataset and Metrics

The SUN-RGBD dataset is a highly comprehensive dataset for understanding indoor scenes, comprising 10,335 pairs of images depicting various indoor scenes such as living rooms, bedrooms, kitchens, bathrooms, and more. A total of 37 different object categories are annotated in this dataset. Among the images, 5285 are designated for training, while 5050 images are reserved for testing purposes.

The NYUDv2 dataset is composed of video sequences capturing various indoor scenes recorded by Microsoft Kinect’s RGB and depth cameras. This dataset comprises 1449 pairs of densely annotated RGB and depth images, covering a total of 40 different object categories. These categories encompass various objects commonly found in indoor scenes, including chairs, tables, TVs, people, and more. The dataset is split into a training set with 795 images and a test set containing the remaining 654 images.

We measure the proposed CFANet with advanced methods according to three measures: mean accuracy (mAcc), pixel accuracy (PixAcc), and mean intersection over union (mIoU).

### 4.2. Implementation Details

CFANet is implemented using the PyTorch2.5.0 framework on two NVIDIA GeForce RTX 4080 GPUs. A ResNet50 pretrained on ImageNet is employed as the backbone network for CFANet. During the training phase, all input images are uniformly cropped to a resolution of 480 × 640 pixels. To enhance the model’s generalization ability, various data augmentation techniques are implemented, including random scaling, random cropping, random horizontal flipping, random brightness adjustment, and random saturation adjustment. For optimization, we utilize a stochastic gradient descent (SGD) algorithm with a momentum value of 0.9 and a weight decay factor of 0.0004 as the optimizer. The learning rate is adjusted using a polynomial decay strategy, with the polynomial exponent set to 0.9 and the initial learning rate set to 0.009. Considering the characteristics of different datasets, specific training epochs and batch sizes are set as follows: for the NYUDv2 dataset, the training process spans 250 epochs with each batch containing six images; for the SUN-RGBD dataset, the training process extends over 200 epochs with each batch comprising four images. The cross-entropy loss function is employed as the network’s loss function.

### 4.3. Comparison with Other Methods

Table 1 summarizes recent semantic segmentation research findings on the NYUDv2 dataset, encompassing multiple state-of-the-art models. Our evaluation of the proposed CFANet on the same dataset demonstrates clear performance advantages over existing approaches. The experimental results demonstrate that CFANet achieves an outstanding performance on the key metric of mIoU, surpassing the second-best model FCINet [32] by approximately 2.16%. This substantial margin effectively validates the effectiveness of the newly introduced modules in CFANet. It is noteworthy that, compared to SCN [33] with ResNet-152 as the backbone network, CFANet exhibits a superior performance in mIoU, suggesting that even in a relatively simple network structure, CFANet can enhance the accuracy of semantic segmentation. Compared to TSTNet [34] with ResNet-34 as the backbone network, CFANet achieves improvements of 7.76%, 5.71%, and 4.97% in mIoU, mACC, and PixACC, respectively, highlighting the effectiveness of applying residual connection strategies in multiple modules of CFANet.

The effectiveness of CFANet is validated on the larger-scale SUN-RGBD dataset, as shown in Table 2. Specifically, compared to FCINet with ResNet-50 as the backbone network, CFANet achieves a 2.35% improvement in mIoU. Notably, when compared to the models in Table 2 employing ResNet-101 as the backbone network, this network still achieves a superior segmentation performance despite utilizing a relatively shallow feature extraction network. This demonstrates CFANet’s excellent capability in handling large-scale datasets, providing strong support for its widespread application in real-world scenarios.

Although CFANet achieves a 53.86% and 51.85% mIoU on the NYUDv2 and SUN-RGBD datasets, respectively, it is necessary to acknowledge that certain advanced models demonstrate a superior performance in terms of mIoU. As shown in Table 1 and Table 2, AESeg [40] obtains a 59.7% and 57.7% mIoU on these datasets, representing improvements of 5.84% and 5.85% over CFANet. Notably, CFANet reduces the number of parameters (Params) and computational complexity (GFLOPs) by 81.8 and 54.6 compared to AESeg, respectively. This performance–efficiency balance indicates that CFANet achieves significant computational resource optimization through moderate mIoU degradation. The experimental results further validate the effectiveness of our proposed RGB-D cross-modal interaction strategy and adaptive dynamic alignment of cross-modal features. These mechanisms effectively address the semantic gap in RGB-D semantic segmentation tasks while maintaining a balanced trade-off between mIoU performance and computational cost.

### 4.4. Visualization Results

**Figure 8:**
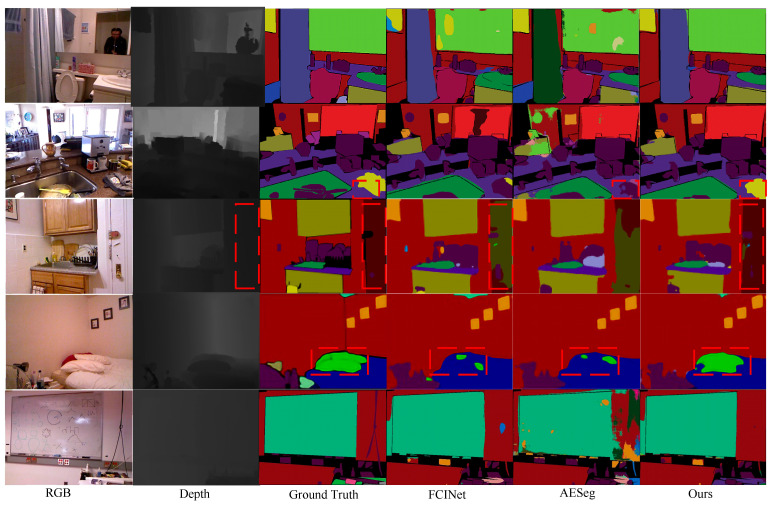
Visualization results on NYUDv2.

To visually showcase the significant advancements of CFANet in semantic segmentation tasks, the visualization results on the NYUDv2 dataset are provided, as shown in Figure 8. A comparison is made with two advanced models.

In the first row, noise is present in the feature extraction of “mirror” predictions from both RGB and depth images. CFANet, by integrating contextual information, accurately achieves the segmentation of the “mirror”. In the second row, compared to AESeg utilizing asymmetric convolution, CFANet more accurately segments the object within the red dashed box, highlighting the effectiveness of CFANet’s combination of asymmetric convolution and dilated convolution. In the third row, FCINet attempts to enhance the network’s segmentation accuracy for objects of different scales using spatial pyramid pooling. However, it performs poorly in segmenting large-scale objects (the object within the red dashed box). In contrast, CFANet achieves relatively advanced results by extracting significant single-modal features from the channel and spatial dimensions of the depth map and interacting with the feature map of the RGB image. In the fourth row, the object within the red dashed box has a similar color to the bed. Both FCINet and AESeg fail to segment this object effectively, while CFANet’s segmentation result is superior. This is attributed to the appropriate feature extraction modules applied to RGB images and depth maps. These examples highlight the superiority of CFANet in semantic segmentation tasks, emphasizing its effectiveness in reducing the semantic gap between RGB and depth images and minimizing information loss.

### 4.5. Ablation Studies

The CFANet proposed by us comprises several modules, including BFEM, DFEM, AFCFM, SCM and FBH. To validate the effectiveness of each module, we conducted extensive ablation experiments on NYUDv2.

#### 4.5.1. Validate BFEM and DFEM

**Figure 9:**
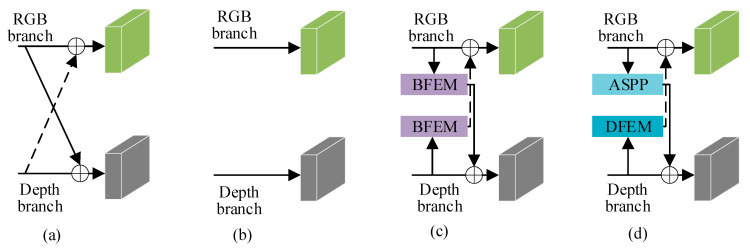
Variants of feature interact. (**a**) Pixel-wise addition for RGB and depth feature interaction. (**b**) The feature interaction between the RGB branch and the depth branch is canceled. (**c**) Using BFEM instead of DFEM to achieve feature re-extraction of the depth branch. (**d**) Using ASPP instead of BFEM to achieve feature re-extraction of the depth branch.

In this section, we designed four different variants of feature interaction, as illustrated in Figure 9, and obtained experimental results for each variant on the NYUDv2 dataset. The results are recorded in Table 3. By comparing the experimental outcomes of each variant, we substantiate the effectiveness of our research approach.

**Figure 10:**
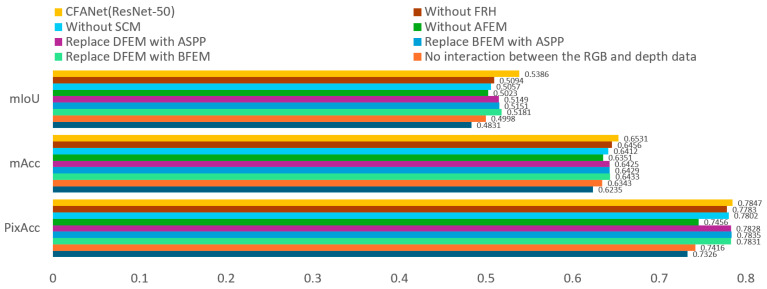
Ablation study for various components of CFANet conducted on the NYUDv2 dataset.

As shown in Figure 9a, we remove the BFEM and DFEM modules from CFANet (ResNet-50). The experimental results in Figure 10 show a 5.55% decrease in mIoU compared to CFANet (ResNet-50). Figure 9b presents a variant without feature interaction between the RGB branch and depth branch. The mIoU drops by 3.88%, with a smaller decrease than the strategy in Figure 9a. This indicates that a semantic gap exists between RGB feature maps and depth feature maps. Direct addition of these features leads to performance degradation. Therefore, a design is required to separately extract modality features, as proposed in this work.

As shown in Figure 9c, the AFCFM module is removed from CFANet (ResNet-50). The experimental results in Figure 10 show a 3.12% decrease in mIoU compared to the complete version of CFANet (ResNet-50). This result indicates that the AFCFM effectively addresses the semantic discrepancy between RGB and depth features. As shown in Figure 9d, the removal of the SCM and FRH modules results in a 2.44% drop in mIoU, thereby demonstrating the substantial contribution of the multi-scale feature fusion strategy to the improvement of segmentation performance.

As shown in Figure 9d, the BFEM module in the RGB branch is replaced with an ASPP module. The experimental results in Figure 10 show a 2.35% decrease in mIoU compared to the complete version of CFANet (ResNet-50). Since BFEM is an improvement over ASPP, this result validates the effectiveness of our improvement strategy.

Similar to Figure 9d, the DFEM module in the depth branch is replaced with an ASPP module. The experimental results in Figure 10 show a 2.37% decrease in mIoU compared to the complete version of CFANet (ResNet-50). The effectiveness of the DFEM module is confirmed.

#### 4.5.2. Validate AFEM, SCM, and FRH

As illustrated in Figure 10, the AFEM module is replaced with element-wise summation, the SCM module is also substituted with element-wise summation, and the FRH component is removed. The mIoU decreased by 3.63%, 3.11%, and 2.92% compared to CFANet (ResNet-50), respectively. This indicates that AFEM, SCM, and FRH in CFANet (ResNet-50) are effective.

#### 4.5.3. Validate Backbone Network

To evaluate the impact of different backbone networks on the performance of CFANet, this paper retains all modules of CFANet and only replaces the backbone network. As shown in Table 3, this paper selects VGG16, ResNet-18, ResNet-34, and ResNet-50 as alternatives for the backbone network. The experimental results indicate that CFANet with ResNet-50 as the backbone network significantly outperforms the other three variants in terms of mIoU, validating the rationality of using ResNet-50 as the backbone network for CFANet. In contrast, CFANet with ResNet-18 as the backbone network shows a decline in mIoU, which may be due to the limited representation ability of shallower networks for key features, indicating that it is not advisable to excessively reduce network depth to minimize model parameters. Similarly, when ResNet-101 serves as the backbone network for CFANet, a slight decline in mIoU is observed, which may be attributed to the loss of crucial feature information resulting from the indiscriminate increase in the number of network layers. Overall, the experiments indicate that ResNet-50 is identified as the most suitable backbone network for CFANet.

### 4.6. Model Quantization and Generalization Performance Evaluation

In this subsection, the module parameters in CFANet and their contribution to improving the mIoU of the baseline model are analyzed. We replace each designed module in CFANet with element-wise addition operations, resulting in the modified network serving as the baseline model. The baseline model achieves an mIoU of 37.39%. Furthermore, the performance of CFANet on the CamVid dataset is validated.

As shown in Table 4, we sequentially add BFEM, DFEM, AFCFM, SCM, and FRH to the baseline model, achieving mIoU improvements of 2.31%, 3.24%, 4.51%, 3.11%, and 2.92%, respectively. The proportion of parameters occupied by each module in CFANet, as well as their contributions to computational complexity, are further analyzed. Differing from the experimental methodology in the Ablation Studies Section, our approach incrementally adds modules to the baseline model. The results demonstrate the effectiveness of the proposed modules.

To further verify the cross-scenario generalization capability of CFANet, experiments on the outdoor road scene dataset CamVid are conducted. The corresponding experimental results are shown in Table 5. This table presents the performance of the proposed method under pure RGB input conditions on the CamVid dataset. Through comparative analysis with existing methods, the effectiveness of the proposed approach in outdoor scenarios is demonstrated by the experimental results. However, it must be acknowledged that there remains room for improvement for CFANet when compared to models specifically tailored for outdoor environments.

## 5. Conclusions

A semantic segmentation network named CFANet is proposed to address the issues of semantic gaps and information loss in RGB-D semantic segmentation. The semantic gap between different modalities is narrowed through the combined application of a differentiated feature extraction scheme, an appropriate interaction strategy, and an effective multi-modal fusion method. The issue of information loss is mitigated through multi-scale feature map fusion and feature refinement methods. CFANet provides a comprehensive technical approach for analyzing semantic segmentation problems from multiple angles. Experimental results on the NYUDv2, SUN-RGBD, and CamVid datasets validate the effectiveness of CFANet. However, there is still room for optimization in terms of the number of parameters and generalization capability of CFANet. In the future, we will combine techniques such as pruning, knowledge distillation, and matrix decomposition to reduce the model’s parameter count and improve its generalization ability, thereby facilitating the deployment of CFANet on edge artificial intelligence devices.

## Figures and Tables

**Table 1:** Quantitative comparison on NYUDv2 dataset.

Models	Backbone	Params	GFlops	PixAcc	mAcc	mIoU
TSNet [34]	ResNet-34	65.3	67.8	73.50	59.6	46.1
DensnMTL [35]	ResNet-101	106.8	98.6	-	-	40.84
SCN [33]	ResNet-152	98.5	100.7	-	-	50.7
TCANet [36]	ResNet-50	-	-	71.3	60.3	47.8
ESANet [37]	ResNet-50	75.3	83.5	74.42	59.27	46.79
CANet [38]	ResNet-50	86.5	83.9	77.1	64.6	50.9
Link-RGBD [39]	ResNet-50	-	-	76.8	59.6	49.5
AESeg [40]	ResNet-50	24.13	27.63	77.0	-	50.7
Z-ACN [41]	ResNet-50	68.2	70.4	75.88	63.55	50.05
FCINet [32]	ResNet-50	-	-	75.9	63.2	51.7
AESeg [40]	ResNet-50	163.4	141.8	-	-	57.7
Ours (CFANet)	ResNet-50	81.6	87.2	78.47	65.31	53.86

**Table 2:** Quantitative comparison on SUN-RGBD dataset.

Models	Backbone	Params	GFlops	PixAcc	mAcc	mIoU
CANet [38]	ResNet-101	86.5	83.9	72.5	60.5	49.3
PDCNet [42]	ResNet101	-	-	72.4	-	49.2
SGNet [2]	ResNet-101	64.7	108.5	71.0	-	47.5
CGBNet [43]	ResNet-101	-	-	72.3	-	48.2
CMX-B2 [44]	MiT-B2	66.6	67.6	72.8	-	49.7
ESANet [37]	ResNet-34	61.6	62.4	-	-	48.2
Link-RGBD [39]	ResNet-50	-	-	73.1	53.5	48.4
ACNet [45]	ResNet-50	76.6	66.7	-	-	48.1
ESANet [37]	ResNet-50	75.3	83.5	-	-	48.3
FCINet [32]	ResNet-50	-	-	72.6	60.9	49.5
AESeg [40]	ResNet-50	163.4	141.8	-	-	52.8
Ours (CFANet)	ResNet-50	81.6	87.2	83.62	64.53	51.85

**Table 3:** Experimental results of CFANet based on different backbones.

Backbone	PixAcc	mAcc	mIoU
VGG16	75.17	61.79	48.75
ResNet18	76.75	63.58	50.52
ResNet101	78.02	63.66	52.08
ResNet50	78.47	65.31	53.86

**Table 4:** Quantitative analysis of modules on the NYUDv2 dataset.

Modules	mIoU Contribution	Params	GFlops
BFEM	+2.31	19%	20%
DFEM	+3.24	11%	12%
AFCFM	+4.51	26%	33%
SCM	+3.11	2%	5%
FRH	+2.92	8%	11%

**Table 5:** CFANet experimental results on the CamVid dataset.

Method	mIoU
DMPNet [46]	69.2
LETNet [47]	70.5
CACNet [48]	74.6
BSSNet-T [49]	79.5
CFANet (ResNet-50)	76.1

## Data Availability

The SUN-RGBD dataset used in this study can be obtained from the website https://rgbd.cs.princeton.edu/ (accessed on 16 January 2025), and the NYUv2 dataset can be obtained from the website https://cs.nyu.edu/~fergus/datasets/nyu_depth_v2.html (accessed on 16 January 2025).
